# Anxiety and posttraumatic stress in post-acute sequelae of COVID-19: prevalence, characteristics, comorbidity, and clinical correlates

**DOI:** 10.3389/fpsyt.2023.1160852

**Published:** 2023-06-02

**Authors:** Stephen J. Ferrando, Sean Lynch, Nicole Ferrando, Rhea Dornbush, Sivan Shahar, Lidia Klepacz

**Affiliations:** ^1^Department of Psychiatry and Behavioral Sciences, New York Medical College, Valhalla, NY, United States; ^2^Department of Psychiatry, Westchester Medical Center Health System, Valhalla, NY, United States; ^3^Department of Psychiatry, Icahn School of Medicine at Mount Sinai, Mount Sinai Beth Israel, New York, NY, United States; ^4^Department of Psychiatry, Icahn School of Medicine at Mount Sinai, Mount Sinai Hospital, New York, NY, United States

**Keywords:** anxiety, PTSD, COVID-19, post-acute sequelae of SARS-CoV-2 infection, cognitive complaints

## Abstract

**Background:**

Anxiety and post-traumatic stress symptoms have been reported in association with acute and post-acute sequelae of COVID-19 (PASC).

**Purpose:**

This study aimed to document the cross-sectional prevalence, characteristics and clinical correlates of anxiety and post-traumatic stress in a study of neuropsychiatric sequelae of COVID-19.

**Method:**

75 participants recruited from a post-COVID-19 recovery program and the community were assessed for sociodemographic, medical, psychiatric, and neurocognitive symptoms and performance. The generalized anxiety questionnaire-7 (GAD-7) and post-traumatic stress disorder questionnaire for DSM5 (PCL5) were utilized to measure anxiety and PTSD symptoms. Established cutoff scoring for the GAD-7 and algorithm-based scoring of the PCL5 were utilized to determine clinically significant anxiety symptoms and PTSD, respectively.

**Results:**

The cohort was 71% female, 36% ethnic minority, with the main age of 43.5 years, 80% employment, 40% with the prior psychiatric treatment history and 2/3 seeking post-COVID care for PASC. Clinically significant anxiety symptoms were found in 31% and PTSD was found in 29% of the cohort. Nervousness and excessive worry were the most prominent anxiety symptoms, while changes in mood/cognition and avoidance were most frequent in PTSD. There was a high degree of comorbidity between clinically significant anxiety symptoms, PTSD, depression and fatigue. In logistic regression, acute COVID illness severity, prior psychiatric history, and memory complaints (but not objective neuropsychological performance) predicted clinically significant anxiety symptoms and/or PTSD.

**Conclusion:**

Clinically significant anxiety and PTSD are found in approximately 1 of 3 individuals after COVID-19 infection. They are highly comorbid with each other as well as with depression and fatigue. All patients seeking care for PASC should be screened for these neuropsychiatric complications. Symptoms of worry, nervousness, subjective changes in mood, and cognition as well as behavioral avoidance are particularly important targets of clinical intervention.

## Introduction

The COVID-19 pandemic has generated a significant amount of anxiety, for individuals and for society. In the early stages of the pandemic, fear of contagion, lockdown orders and economic concerns generated considerable anxiety, regardless of COVID-19 infection *per se*. Two years into the pandemic, the multi-system post-acute sequelae of COVID-19 (PASC) have been extensively reported (1). Among these, neuropsychiatric sequelae, including mental health symptoms, fatigue, and neuropsychological complaints (“brain fog”) are highly prevalent. In a meta-analysis of PASC symptoms reported greater than 3 months after the onset of COVID-19 infection, approximately 23% (range 14–32%) of individuals were affected by anxiety, 17% by depression and 31% by sleep disturbances (2). Importantly, patients with more severe COVID illness, who had been hospitalized and in intensive care had a higher prevalence of these neuropsychiatric symptoms compared to those with less severe illness.

A systematic review of neuropsychiatric symptoms of PASC found that anxiety symptoms were assessed in multiple studies, primarily in hospitalized cohorts followed approximately 6 months after discharge, with assessment conducted via online survey and clinical interview, employing various validated scales (3). Overall, clinically significant anxiety was reported in 5–30% of patients, with risk factors including COVID illness severity and duration, female gender, and prior psychiatric history. Aside from stressors related to COVID illness and the pandemic, elevated systemic inflammation during acute COVID illness has been postulated to cause or contribute to the development or persistence of neuropsychiatric symptoms in PASC. However, data are limited and early findings inconsistent. For instance, Mazza et al. (4), found that the Systemic Illness Index (SII), but not other inflammatory markers measured during acute COVID-19 hospitalization, was associated with depression and anxiety at 1 month follow-up, but only depression at 3 months. These authors also found that female gender and prior psychiatric history were important predictors of persistent psychiatric symptoms 1–3 months after acute illness. They suggested that psychological factors such social isolation, fear of mortality, contagion and stigma must be considered alongside any biological marker.

Post-traumatic stress has also been documented in the months after COVID-19 illness. In the systematic review cited above, PTSD was reported in 6.5–42.8% of primarily post-hospitalized cohorts (3). There are many aspects of COVID-19 illness and the consequences of the pandemic in general that might serve as traumatic events leading to the development of PTSD. In the context of COVID illness, intensive care unit treatment, prior psychiatric history, pre-COVID traumatic events and depressive and anxiety symptoms at time of acute COVID illness have been found to confer risk for PTSD in some, but not all studies (3).

While anxiety and PTSD symptoms have been reported in the months after COVID-19 infection, further information is needed regarding the prevalence of clinically significant anxiety and PTSD, as well as their symptom characteristics and predictors after acute COVID-19 illness. This information will guide screening, clinical assessment and treatment. In our experience, we have found that anxiety symptoms are important component of the clinical presentation of PASC and that various effects of COVID-19 illness and pandemic-related stress, including occupational problems and social isolation, are traumatic, requiring psychotherapeutic and psychopharmacological treatment (5).

As part of a prospective study of post-acute neuropsychiatric sequelae of COVID-19, we sought to add to the current knowledge-base regarding anxiety symptoms and PTSD in PASC, in order to help guide clinical care. We recruited a cohort of individuals after acute COVID-19, both from the community as well as from an academic hospital-based post-COVID recovery program. Participants were assessed with a detailed neuropsychiatric battery, including validated measures of generalized anxiety and PTSD. We aimed to investigate:

1. What proportion of the sample met criteria for clinically significant anxiety symptoms and PTSD, based on validated criteria?
2. What were the most prevalent individual symptoms of anxiety and which symptoms better differentiate those meeting criteria for clinically significant anxiety compared to those not meeting criteria?
3. Of the 4 PTSD symptom domains (intrusive thoughts, avoidance, alterations in mood and cognition, and hyperarousal), which were the most prevalent and which domains better differentiate those with PTSD compared to those without PTSD?
4. What was the rate of comorbidity of clinically significant anxiety, PTSD, depression and clinically significant fatigue in the cohort?
5. What were significant sociodemographic, medical (COVID-19 severity and other medical comorbidities) and prior psychiatric predictors of clinically significant anxiety and PTSD?

## Methods

This study was conducted at Westchester Medical Center Health System (WMC Health)/New York Medical College (NYMC) in Valhalla, New York. The study was approved by both the Institutional Review Board of NYMC (Protocol #14400) and the WMCHealth Clinical Research Institute. The results presented here were obtained from the baseline assessment of 75 participants who were recruited via flyers, social media, email and word-of-mouth for a longitudinal study investigating the neurocognitive, psychiatric and medical sequelae of COVID-19 infection. A subset of study participants was referred from the WMCHealthPost-COVID-19 Recovery Program after seeking care for “brain fog” and other PASC symptoms. Participants were screened via telephone to determine eligibility for participation by the investigators (SL, SS) based on the following criteria: (1) Age at least 20 years old; (2) Participants who reported prior COVID-19 illness symptoms and either documented positive COVID-19 nasopharyngeal test, or, if nasopharyngeal test was unavailable, documented positive antibody test prior to being vaccinated; (3) recovered from acute COVID-19 infection as per CDC recommendations at the time of assessment (i.e., 10–20 days after symptom onset and 24 h without fever); (4) completed minimum 8th grade education; (5) fluent in English; and (6) capable of signing informed consent. Persons with a prior diagnosis of a major neurocognitive disorder, traumatic brain injury with loss of consciousness, uncorrected visual/hearing deficits, intellectual disability, or unstable psychiatric symptoms were excluded.

Participants met with the study assessors (SL, SS), who were trained to perform and score the assessment battery by co-PI (RD), a board-certified Neuropsychologist, and were supervised by the study PI (SF). During this visit, all risks and benefits were explained to prospective participants, and signed informed consent was obtained. Participants were compensated $40.00 for their time.

### Study measurements and instruments

The primary measures of interest in this study were the Generalized Anxiety Disorder-7 (GAD-7) questionnaire (6) and the Post Traumatic Stress Disorder Checklist for DSM-5 (PCL-5) (7). The GAD-7 is a 7-item scale that rates symptoms of anxiety (nervousness, excessive worry, inability to stop worrying, fear, restlessness, inability to relax and irritability) on a 0 (none) to 3 (severe) scale, with a range of 0–21. A score of ≥10 has a sensitivity of 89% and specificity of 82% for diagnosing generalized anxiety disorder in general medical populations (6), so was used as the cutoff to determine clinically significant anxiety symptoms for this study. In the initial validation study, the GAD-7 scale also performed well (area under the curve, 0.80 to 0.91) as screening tool for panic disorder, social anxiety and PTSD, so an elevated score on the GAD-7 may detect a range of anxiety disorders. Thus, the GAD-7 as utilized in this study reflected anxiety symptoms in general, but was not specific to a particular anxiety disorder *per se*.

The PCL-5 is a 20-item self-report measure that assesses the presence and severity of PTD symptoms (7). Items correspond to the DSM-5 PTSD criteria, and can be categorized into the 4 PTSD symptom domains (Criterion B- re-experiencing, questions 1–5; Criterion C - avoidance, questions 6–7; Criterion D- alterations in mood and cognition, questions 8–14; Criterion E - hyper-arousal, questions 15–20). The PCL-5 has a maximum score of 80. There are multiple mechanisms to score this measure in order to screen for a clinical provisional diagnosis of PTSD. For this study, we chose a criterion-based scoring algorithm, which closely reflects clinical practice (8). In this algorithm, a provisional PTSD diagnosis can be made by treating each item rated as 2 = “Moderately” or higher as a symptom endorsed, then following the DSM-5 diagnostic rule which requires at least: 1 B item (questions 1–5), 1 C item (questions 6–7), 2 D items (questions 8–14), 2 E items (questions 15–20).

The Patient Health Questionnaire-9 (PHQ-9), a 9-item self-administered clinical tool, was used in the provisional diagnosis of depression and the measurement of depression severity (9). The PHQ-9 score ranges from 0 to 27. Analogous to our approach for PTSD, in order to best reflect clinical practice, we employed the criterion-based approach using the PHQ-9 to make a provisional diagnosis of MDD. For this study, provisional major depression was considered likely present if 5 or more (out of the 9) depressive symptoms are present for “more than half of the days” over the previous 2 weeks, with 1 of the symptoms being depressed mood or anhedonia. Thoughts of suicide to any degree are included in the provisional diagnosis, based on the scoring algorithm. Finally, on the final item on the PHQ-9 scale that queries functional difficulty, participants had to endorse that the depressive symptoms had made it *at least* “somewhat difficult” to function, in order to make a provisional diagnosis.

The Endicott Quality of Life Enjoyment and Satisfaction Questionnaire (Q-LES-Q) was used to characterize quality of life in this population (10). The Q-LES-Q queries multiple domains of life enjoyment and satisfaction (e.g., mood, occupational and social functioning). The scale has a maximum of 70, with raw scores converted into a total percentage, with higher percentage scores indicating greater quality of life.

Participants underwent neurocognitive evaluations, and medical, psychiatric, and sociodemographic measures were obtained. Sociodemographic measures included age, gender, ethnicity, relationship status, years of education and current employment. Medical measures included a self-reported medical history, and a detailed history of COVID-19 illness (including symptoms, treatment, hospitalization, and time since diagnosis). COVID-19 symptom severity at peak of acute infection as well as at the time of the study appointment was determined by score on an instrument adapted from published CDC COVID-19 symptoms, assessing severity (absent, mild, moderate, severe) on 11 COVID-19 symptoms (11). Participants were assessed on their daily functioning, using the Lawton-Brody Instrumental Activities of Daily Living Scale (IADL) which measures practical aspects of everyday functioning on a scale of 0–8 (12). Study participants were also evaluated for fatigue using the Chalder Fatigue Scale, an 11-item questionnaire measuring the severity of mental and physical fatigue and is scored from 0 to 33. A cutoff score of >21 is considered clinically significant fatigue (13). Psychiatric assessment also included prior psychiatric treatment and substance use history.

The neurocognitive battery included in this analysis included the Repeatable Battery for the Assessment of Neuropsychological Status (RBANS) Form A, which is a neuropsychological assessment designed to identify and characterize abnormal cognitive decline, and yields both a Total Score as well as sub-scores for five cognitive domains (14). Also included was the Montreal Cognitive Assessment (MoCA)(Used with permission, MoCA Test, Inc., September 9, 2022), a 30-point neurocognitive screening test scored from 0 to 30, with a score below 26 possibly indicative of cognitive impairment (15). Participants additionally completed the Patient Assessment of Own Function (PAOF), which queries subjective cognitive complaints yielding an average score of 0–5 for multiple domains (16). Memory, language/communication and cognitive/intellectual functioning were used for the purposes of this study, as these domains are most frequently endorsed by PASC patients.

Analyses were conducted on the entire sample of 75 participants and then on the two subgroupings based on clinical scoring criteria: “Anxiety” vs. “No Anxiety” and “PTSD” vs. “No PTSD.” All data was analyzed using SPSS software (17), including all descriptive statistics (frequency, mean, and standard deviation); Chi-square for group comparisons on categorical variables and independent sample t-tests were used for group comparisons on continuous variables; Finally, logistic regression was used to identify predictors of combined “Anxiety and/or PTSD,” as the conditions were highly comorbid.

## Results

The overall sample was predominately female (over two thirds), predominately white (approximately two thirds), with over three quarters being currently employed and about two thirds being in a relationship. The average age of the overall sample was 43.5 years, with an average educational attainment equivalent to a bachelor’s degree. The average amount of time since acute COVID-19 infection was 220 days, though with significant variance. The sample was entirely ambulatory, with an average of 1.6 non-COVID comorbidities (primarily obesity, diabetes and hypertension) and a small minority (*N* = 9, 12%) had a history of hospitalization due to COVID-19 infection, but none required intensive care unit care (Table 1).

**Table 1:** Sociodemographic and clinical factors of the total sample of participants (*N* = 75) and subgroups meeting clinical criteria for GAD.

	Total sample (*N* = 75)	Anxiety (*N* = 23, 30.7%)	No anxiety (*N* = 52, 69.3%)	*p*-value
Sociodemographic factors (*N*, %)				
Ethnicity, minority	27 (36%)	8 (34.8%)	19 (36.5%)	0.24
Gender, female	53 (70.7%)	20 (87.0%)	33 (62.2%)	**0.04**
Employment, yes	60 (80%)	18 (78.3%)	42 (80.8%)	0.49
Relationship status, in relationship	49 (65.3%)	11 (47.8%)	38 (73.1%)	0.09
Sociodemographic factors (Mean, SD)				
Appointment 1 age	43.5 (15.0)	44.8 (15.2)	42.9 (14.7)	0.62
Education years	16.0 (2.2)	15.3 (2.0)_	16.3 (2.4)	0.07
Clinical factors (*N*, %)				
Clinic patient	47 (62.7%)	17 (73.9%)	30 (57.7%)	0.18
Psychiatric history, yes	31 (41.3%)	16 (69.6%)	15 (28.8%)	**<0.001**
Psychiatric medications, yes	24 (32.0%)	11 (47.8%)	13 (25.0%)	**0.05**
Substance Use Hx.	12 (16.0%)	4 (17.4%)	8 (15.4%)	0.83
Medical measures (Mean, SD)				
Peak COVID Sx.	16.6 (6.2)	20.8 (5.7)	14.8 (5.5)	**<0.001**
Appointment 1 COVID Sx.	6.4 (4.8)	9.2 (4.7)	5.2 (4.3)	**<0.001**
# Medical Comorbidities	1.6 (1.5)	1.7 (1.5)	1.4 (1.5)	0.43
Days from COVID Dx. to Appt. 1	220.6 (136)*	198.7 (126.7)	230.0 (140.1)	0.36
Chalder	21.7 (7.6)*	26.4 (4.3)	19.6 (7.8)	**<0.001**
IADL	7.5 (1.1)*	7.3 (1.1)	7.6 (1.1)	0.27
Psychiatric measures (Mean, SD)				
GAD-7	7.3 (5.5)	14.3 (3.3)	4.1 (2.8)	**<0.001**
PCL-5	21.6 (15.2)	36.7 (12.7)	15.0 (10.9)	**<0.001**
PHQ-9	10.1 (6.3)	15.2 (3.4)	7.8 (3.1)	**<0.001**
Endicott	57.2% (20.0%)	42.2% (14.3%)	63.8% (18.6%)	**<0.001**
Neurocognitive measures (Mean, SD)				
PAOF memory	2.03 (1.16)	2.90 (0.95)	1.64 (1.03)	**<0.001**
PAOF language	1.56 (1.08)	2.37 (0.91)	1.19 (0.95)	**<0.001**
PAOF cognition	1.70 (1.29)	2.54 (1.23)	1.31 (1.13)	**<0.001**
RBANS, total scaled score**	93.2 (14.6)	89.0 (18.5)	95.1 (2.3)	0.16
MOCA, total**	25.7 (2.7)	25.0 (2.8)	26.0 (2.5)	0.10

* These values were based on *N* = 74 due to missing data.

** RBANS and MOCA subscale scores were not significantly different between GAD diagnostic groups, data not shown. Values in bold are statistically significant.

### Anxiety in the sample

**Figure 1:**
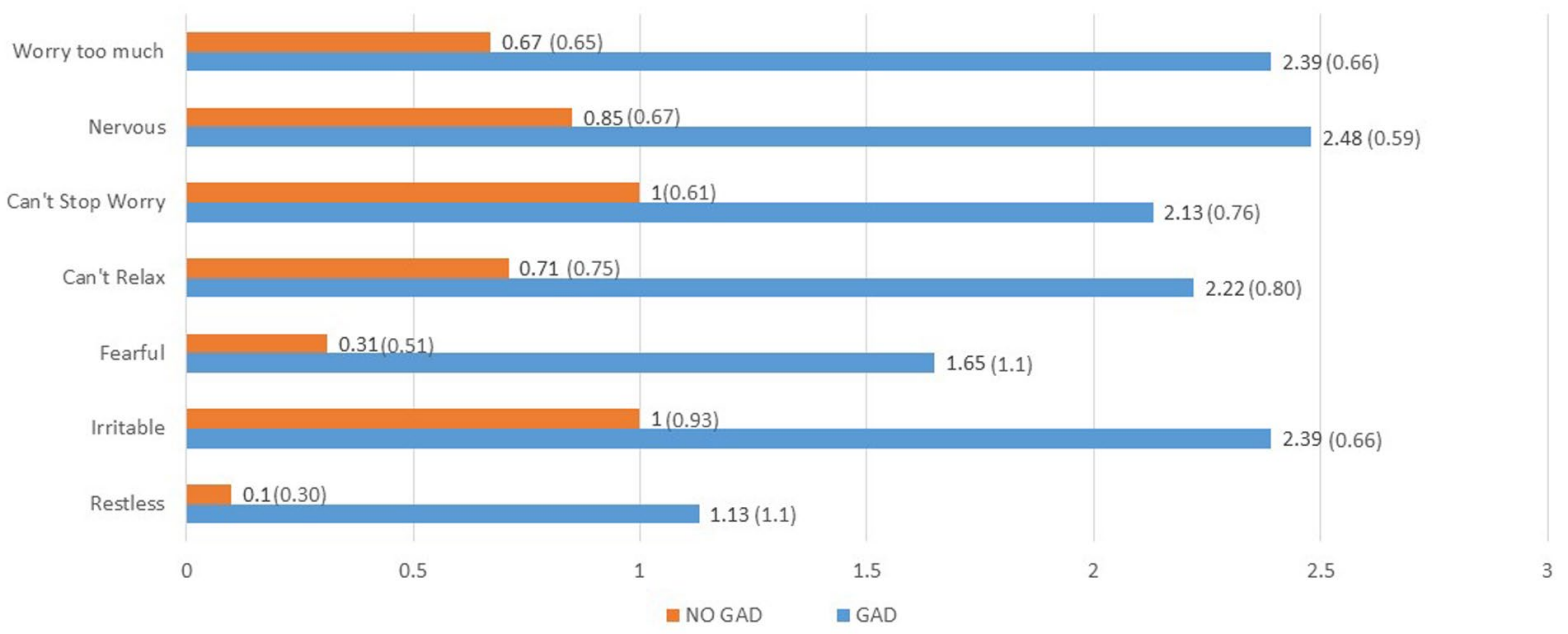
Comparison participants meeting criteria for GAD (*N* = 23) vs. those not meeting criteria (*N* = 52) on mean (sd) value of individual anxiety symptoms, based on greatest to least effect size (Hedges *g*)^1,2^. ^1^All *p* < 0.001. ^2^Hedges *g*: worry too much 2.64; nervous 2.53; can’t stop worrying 2.45; can’t relax 1.97; fearful 1.86; irritable 1.63; restless 1.58.

Using clinical cutoff scoring, 23 (31%) met criteria for clinically significant anxiety (Table 1). Nearly 2 in 3 of them had a prior psychiatric treatment history: all but one of these (15, 65%) reported a prior diagnosis of anxiety disorder and 13 (57%) reported a history of depression. To characterize the symptoms of anxiety experienced by this cohort, the questions on the GAD-7 were examined individually and compared between the Anxiety and the No Anxiety groups, listed in order based on greatest effect size (Hedges g) difference on each item between the groups (Figure 1). The symptoms showing the greatest difference between the Anxiety and No Anxiety groups were excessive worry, nervousness and inability to stop worrying, followed by inability to relax, irritability, fear and restlessness.

Table 1 depicts comparison of Anxiety and No Anxiety groups across measurement domains. The Anxiety group was more likely to be female, but there were no other significant sociodemographic differences. In terms of medical measures, the Anxiety Group had on average a higher COVID symptom severity score both during the acute infection and at the time of the appointment, in addition to higher scores on the Chalder Fatigue Scale.

In terms of psychiatric measures, more participants in the Anxiety Group had a prior psychiatric history, especially in terms of prior diagnosis of anxiety, and were also more likely to be prescribed psychotropic medications (Table 1). The Anxiety Group also differed from the No Anxiety group in all psychiatric measures, including PHQ-9 score that was nearly twice that of the No Anxiety group, as well as significantly higher PCL-5, and lower quality of life Q-LES-Q and IADL scores.

From the standpoint of neurocognitive function, those with anxiety had significantly higher scores on the PAOF subscales of memory, language/communication, and cognitive/intellectual functioning, indicating greater subjective difficulty. However, on neuropsychological testing, there was no significant difference noted between the groups in terms of RBANS Total and subscale scores or MoCA total or subscale scores.

### PTSD in the sample

**Figure 2:**
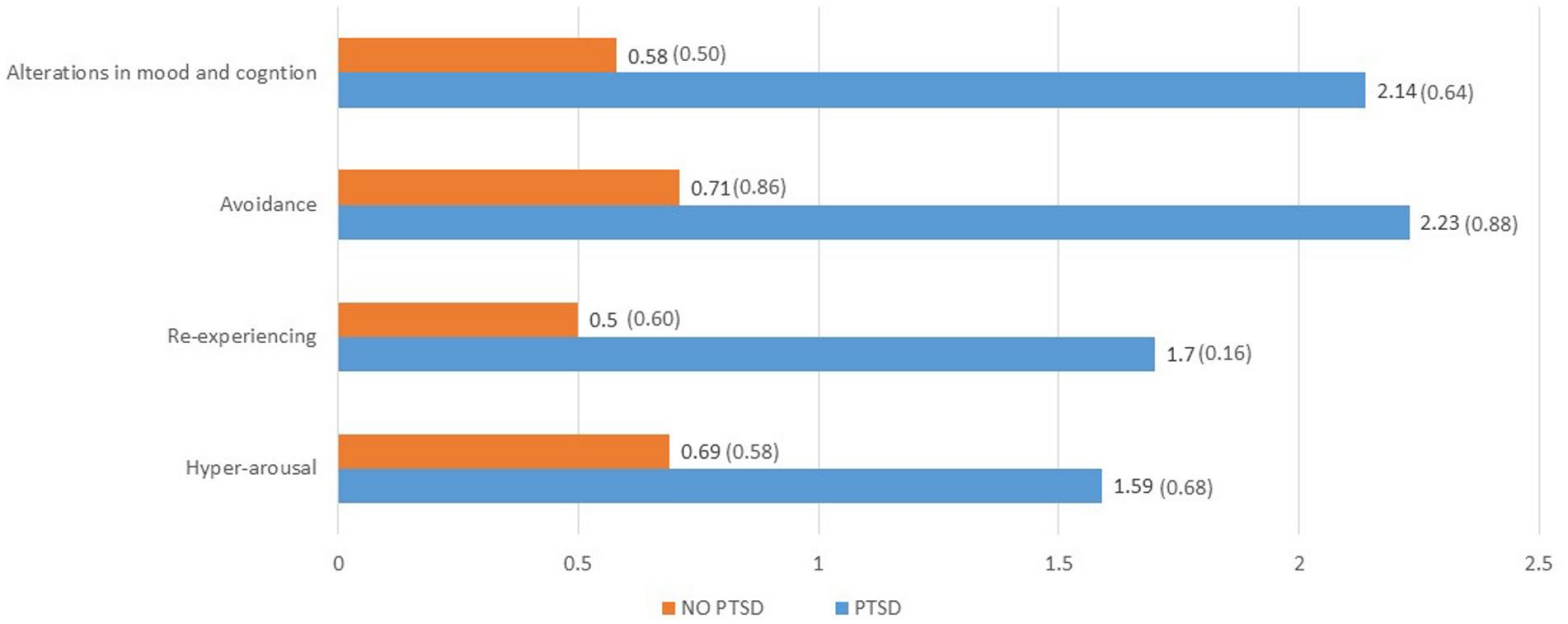
Comparison participants meeting criteria for PTSD (*N* = 22) vs. those not meeting criteria (*N* = 53) on mean (sd) value of each item on four PCL-5 symptom domains, based on greatest to least effect size (Hedges *g*)^1,2^. ^1^All *p* < 0.001. ^2^Hedges *g*: alterations in mood and cognition 2.58; avoidance 1.73; re-experiencing 1.60; hyper-arousal 1.45.

Using clinical criterion-based scoring for PTSD, 22 (29%) met criteria for PTSD. Similar to anxiety, nearly 3 in 4 participants meeting PTSD criteria had a prior psychiatric history. However, in contrast to the Anxiety group, only 2 (9%) reported a prior diagnosis of PTSD. Twelve (55%) reported a prior diagnosis of depression. To characterize the PTSD symptom clusters experienced by this cohort, the questions on the PCL-5 were categorized into the 4 symptom domains of re-experiencing, hyper-arousal, avoidance and alterations in cognition and mood, and an average score for each domain was obtained by dividing by the number of questions in each domain. In Figure 2, the PTSD symptom domains are compared between the clinical PTSD group and the No PTSD group, and are listed in order based on greatest to least effect size (Hedges g) between the groups. The symptoms showing the greatest effect size difference between the PTSD and No PTSD groups were alterations in mood and cognition and avoidance, followed by re-experiencing and hyper-arousal.

Table 2 depicts comparison of PTSD and No PTSD groups across measurement domains. There were no significant sociodemographic differences. In terms of medical measures, the PTSD Group had on average a higher COVID symptom severity score both during the acute infection and at the time of the appointment, in addition to higher scores on the Chalder Fatigue Scale. The PTSD group were also more likely to be seeking clinical care for PASC, and they reported more limitations in IADL.

**Table 2:** Sociodemographic and clinical factors of the total sample of participants (*N* = 75) and subgroups meeting clinical criteria for PTSD.

	Total sample (*N* = 75)	PTSD (*N* = 22, 29%)	No PTSD (*N* = 53, 71%)	*p*-value
Sociodemographic factors (*N*, %)				
Ethnicity, minority	27 (36%)	6 (27.3%)	21 (39.6%)	0.31
Gender, female	53 (70.7%)	17 (77.0%)	36 (67.9%)	0.42
Employment, yes	60 (80%)	16 (72.7%)	44 (83.0%)	0.27
Relationship status, In relationship	49 (65.3%)	14 (63.6%)	35 (66.0%)	0.84
Sociodemographic factors (Mean, SD)				
Appointment 1 age	43.5 (15.0)	47.2 (15.0)	42.0 (14.8)	0.17
Education years	16.0 (2.2)	15.6 (2.0)_	16.2 (2.3)	0.26
Clinical factors (*N*, %)				
Clinic patient	47 (62.7%)	18 (81.8%)	29 (54%)	**0.03**
Psychiatric history	31 (41.3%)	13 (59%)	18 (31.6%)	**0.04**
Psychiatric medications, yes	24 (32.0%)	10 (45.4%)	14 (26%)	0.11
Substance use Hx.	12 (16.0%)	4 (18.2%)	8 (15.1%)	0.74
Medical measures (Mean, SD)				
Peak COVID Sx.	16.6 (6.2)	21.5 (4.7)	14.6 (5.6)	**<0.001**
Appointment 1 COVID Sx.	6.4 (4.8)	9.6 (5.3)	5.1 (3.8)	**<0.001**
# Medical comorbidities	1.6 (1.5)	2.0 (1.5)	1.4 (1.5)	0.06
Days from COVID Dx. to Appt. 1	220.6 (136)*	236.5 (130.1)	209.8 (140.8)	0.22
Chalder	21.7 (7.6)*	26.6 (4.0)	19.7 (7.9)	**<0.001**
IADL	7.5 (1.1)*	6.8 (1.6)	7.8 (0.80)	**0.006**
Psychiatric measures (Mean, SD)				
GAD-7	7.3 (5.5)	12.6 (4.8)	5.3 (4.1)	**<0.001**
PCL-5	21.6 (15.2)	40.4 (8.9)	13.8 (9.2)	**<0.001**
PHQ-9	10.1 (6.3)	15.9 (4.6)	7.7 (5.2)	**<0.001**
Endicott	57.2% (20.0%)	41.6% (15.4%)	67.0% (18.1%)	**<0.001**
Neurocognitive measures (Mean, SD)				
PAOF memory	2.03 (1.16)	2.90 (1.0)	1.68 (1.02)	**<0.001**
PAOF language	1.56 (1.08)	2.33 (0.95)	1.23 (0.96)	**<0.001**
PAOF cognition	1.70 (1.29)	2.66 (1.27)	1.29 (1.07)	**<0.001**
RBANS, total scaled score**	93.2 (14.6)	90.1 (17.2)	94.5 (13.4)	0.23
MOCA, total**	25.7 (2.7)	24.9 (2.3)	26.0 (2.7)	0.06

* These values were based on *N* = 74 due to missing data.

** RBANS and MOCA subscale scores were not significantly different between groups, data not shown. Values in bold are statistically significant.

In terms of psychiatric measures, the PTSD group was significantly more likely to have a prior psychiatric history than the No PTSD group, and they were nearly twice as likely to be on psychotropic medications (although technically non-significant with *p* = 0.06). The PTSD Group also differed from the No PTSD group in all psychiatric measures, including PHQ-9 score that was on average nearly twice that of the No PTSD group, GAD-7 score nearly 3 times higher, and significantly lower quality of life.

From the standpoint of neurocognitive function, those meeting criteria for PTSD had significantly higher scores on the PAOF subscales of memory, language/communication, and cognitive/intellectual functioning, indicating greater subjective difficulty. However, on objective neuropsychological testing, there was no significant difference noted between the groups in terms of RBANS Total and subscale scores or MoCA total or subscale scores.

### Comorbidity of anxiety, PTSD, depression, and clinical fatigue

There was a high degree of comorbidity among the four clinically significant disorders, anxiety, PTSD, depression and fatigue (Table 3). Nearly two thirds of those who met criteria for anxiety had a provisional diagnosis of PTSD, while upwards of three fourths met criteria for depression. For PTSD, the degree of comorbidy was similar to anxiety. Clinically significant fatigue was almost universal in those meeting anxiety and PTSD criteria, exceeding 90%.

**Table 3:** Comorbidity of participants who met criteria for GAD and/or PTSD.

	Anxiety (*N* = 23)	PTSD (*N* = 22)
Anxiety criteria on GAD-7 (*N*, %)	-	16, 72.7%
PTSD criteria on PCL-5 (*N*, %)	14, 60.9%	-
PHQ-9 depression diagnosis (*N*, %)	17, 73.9%	18, 77.3%
Clinical fatigue (Chalder) (*N*, %)	21, 91.3%	20, 91.0%

### Clinical predictors of anxiety and/or PTSD in the sample

Because of the high comorbidity between anxiety and PTSD, the two groups were collapsed into an “Anxiety and/or PTSD” variable. Backwards stepwise logistic regression was conducted to determine clinical predictors based on variables that differed significantly in one or more of the Anxiety vs. No anxiety or PTSD vs. No PTSD comparisons from Tables 1, 2. The overall regression model (Table 4) was significant (Chi-square = 37.85, df = 3, *p* < 0.001), correctly categorizing 80% of cases with - and 87% of cases without - Anxiety and/or PTSD, respectively. In the model, COVID Symptom Total Score during the time of acute illness, prior psychiatric history, and subjective memory complaints on PAOF were independent predictors of Anxiety and/or PTSD. Variables excluded by the model included gender, number of medical comorbidities, current COVID-19 symptoms, and status as a clinical patient seeking post-COVID care. Individual regression models utilizing the same independent variables to predict anxiety and PTSD separately yielded the same significant predictors (data not shown).

**Table 4:** Multivariate logistic regression with backward elimination predicting anxiety and/or PTSD versus no anxiety and/or PTSD.^1^

Variable	Odds ratio	Wald	B	95% Confidence interval (lower bound)	95% Confidence interval (upper bound)	p-value
Peak COVID symptom score	1.21	7.85	0.19	1.06	1.37	0.005
Prior psychiatric history	4.40	5.09	1.48	1.22	15.92	0.02
PAOF memory	2.23	6.59	0.80	1.21	4.13	0.01
Gender	Removed by backwards stepwise elimination					
Number of medical comorbidities						
Clinical Patient						
Current COVID symptom score						

The model for the remaining three predictors (PAOF memory, Peak COVID Symptom Score, and prior psychiatric history) was significant (−2log likelihood = 61.25, Chi Square = 37.85, df = 3, *p* < 0.001).

1 In order to determine predictors of anxiety and/or PTSD, a backward stepwise variable selection logistic regression model was developed. The procedure excluded four of the predictors.

## Discussion

The results of this study indicate that a substantial proportion of individuals have clinically significant anxiety (31%) and PTSD (29%) 6–8 months after acute COVID-19. The primary strength of the study is that it included in-person, detailed neuropsychiatric assessment, allowing for comparison of individuals who had clinically significant anxiety as well as PTSD across sociodemographic, medical, psychiatric and neurocognitive domains.

The rates of anxiety and PTSD found in this study are at the upper end of prevalence ranges documented in prior systematic reviews of studies in hospitalized cohorts, despite the fact that this cohort consisted largely of individuals with mild to moderate acute COVID-19 illness (2, 3). This may be accounted for by the fact that nearly two thirds of this cohort were seeking care for PASC, with higher degrees of persistent neuropsychiatric symptoms.

There are several important issues raised by these findings that have clinical relevance. First, the study identified the most prevalent symptoms of anxiety and PTSD that could serve as a focus of treatment in individuals with PASC. Among those with anxiety, excessive worry, inability to stop worrying and nervousness were the most prevalent and severe symptoms, and these symptoms most differentiated the Anxiety from the No Anxiety groups. These cognitive symptoms of anxiety superseded the somatic symptoms. We did not assess for the sources of worry and nervousness in the participants, however, it is essential to identify these factors in order to address them clinically.

As for PTSD, the domains of alteration in mood and cognition as well as avoidance were more prevalent than re-experiencing and hyper-arousal. This is a similar pattern to anxiety, where cognitive and behavioral symptoms better differentiate clinically relevant PTSD compared to the somatic symptoms. Given the high degree of comorbidity of anxiety and PTSD with clinical depression in this cohort, it is not surprising that these cognitive/affective and behavioral symptoms predominate. Taken together, the results for both anxiety and PTSD suggest that psychotherapeutic interventions, such as cognitive behavioral therapy, could specifically focus on identifying and diminishing the sources of worry, nervousness, negative emotions and cognitions as well as the tendency toward social avoidance in this population.

It is also important to recognize that clinically significant anxiety, PTSD, depression and fatigue frequently co-occur after COVID-19, ranging from 60 to 94% comorbidity. If any one of these disorders is identified, the others should be assessed. It appears that clinical fatigue almost always accompanies anxiety and PTSD, but is not assessed in the GAD-7 or PCL-5. Similarly, depression is not assessed in these measures. Based on these findings, the authors recommend that the GAD-7, PCL-5, PHQ-9, and Chalder Fatigue Scale be utilized to screen patients presenting to primary care or post-COVID clinical programs.

In group comparisons, there were multiple important differences between the anxiety and PTSD groups compared to those without anxiety and PTSD. Anxiety, but not PTSD, was significantly more prevalent in women, but no other sociodemographic differences were identified. Both disorders had higher degrees of acute and current symptoms of COVID-19 (in addition to fatigue, already discussed). In addition, those with PTSD were more likely to be seeking clinical care for PASC compared to their counterparts without PTSD.

There was a highly relevant pattern identified when comparing the clinical and non-clinical anxiety and PTSD groups on subjective cognitive complaints and objective neuropsychological performance. Those with anxiety and PTSD consistently endorsed more subjective difficulties in memory, language and higher cognitive (executive) functions, but they did not differ from their counterparts on any measure of neuropsychological performance. Thus, anxiety and PTSD are associated with perceived, but not actual, cognitive deficits. This has important clinical implications. While individuals who meet criteria for anxiety and PTSD post-COVID-19 may indeed have cognitive dysfunction, the degree of perceived dysfunction may be amplified. While cognitive screening with instruments such as the MOCA is suggested in patients with PASC, screening instruments may be unable to detect mildly low neuropsychological function (18).

In terms of independent predictors of anxiety and/or PTSD in this cohort, severity of COVID illness in the acute stage, prior psychiatric history and subjective memory complaints were all significant independent predictors. Acute COVID illness severity, particularly hospitalization and ICU treatment, have been associated with anxiety and other neuropsychiatric symptoms post-COVID-19 in hospitalized patients (2, 3). These findings suggest that acute COVID illness severity increases the risk for clinical anxiety and PTSD even in individuals with mild to moderate illness. Prior history of psychiatric treatment is also a strong predictor of anxiety and/or PTSD post-COVID-19, and likely constitutes a vulnerability for worsened symptoms. Prior psychiatric history should always be included in clinical assessment, in order to stratify risk for these disorders. Finally, subjective cognitive complaints, particularly memory difficulties, independently predicted anxiety and/or PTSD in this cohort. Given the lack of objective evidence of diminished neuropsychological performance in individuals with anxiety and/or PTSD, it is more likely that the presence of these disorders is amplifying the perception of cognitive difficulty.

This study has important limitations. The cohort is relatively small, and statistical power limited, particularly when comparing those meeting criteria for anxiety and PTSD to those without the disorder and when constructing a logistic regression to identify predictors. Nonetheless, this limitation is mitigated by the detailed and multi-domain assessment battery, including validated assessments of anxiety, PTSD and other neuropsychiatric variables. Another important limitation is that individuals were not assessed clinically prior to COVID-19 to determine pre-COVID levels of anxiety and PTSD symptoms. Further, they were not assessed with a formal diagnostic interview to validate the provisional diagnosis made via cutoff and criterion-based scores on the GAD-7 and PCL-5. Further, the GAD-7 may effectively screen for multiple anxiety disorders (6), however, such specificity could not be achieved in this study. The timeframe for assessment of COVID-related PTSD may have been too brief in 3 participants (none of whom had clinically significant anxiety or PTSD), as they were assessed between 20 and 30 days after acute illness. Another limitation is that specific sources of worry and traumatic events that might have led to the provisional diagnosis of anxiety and/or PTSD were not ascertained. While not feasible in the present study, such characterization is essential in clinical practice. In addition, causal relationships among the variables could not be ascertained using the cross-sectional study design. The cohort may be regarded as biased toward inclusion of individuals seeking post-COVID care for PASC, and therefore may have elevated prevalence of anxiety and PTSD after COVID-19 illness. Another source of bias may have existed in the retrospective rating of acute COVID-19 symptom severity, where symptoms of anxiety, PTSD or other forms of distress may have magnified symptom reports. Regardless of these limitations, the results obtained in the study are consistent with the authors’ clinical experience in assessing and treating neuropsychiatric sequelae of COVID-19 in an outpatient psychiatric program (5).

In conclusion, anxiety and PTSD are prevalent in the setting of PASC. These disorders have multiple important clinical correlates, particularly prior psychiatric history, heightened acute COVID-19 symptoms and perceived cognitive complaints. It is important that further etiological (biological, psychological, and social) as well as treatment research, both psychotherapeutic and psychopharmacological, be dedicated to anxiety and PTSD in PASC. Screening patients at the time of acute illness, including validated assessments, such as those utilized in the current study, and careful attention to prior psychiatric history, will assist in identifying patients at risk for the continuation or development of anxiety and PTSD after acute COVID-19 illness. Finally, clinical populations of PASC patients should be screened for these disorders in order to appropriately target treatment interventions.

## Data availability statement

The raw data supporting the conclusions of this article will be made available by the authors, without undue reservation.

## Ethics statement

The study involved human participants and was reviewed and approved by New York Medical College Institutional Review Board and Westchester Medical Center Health System Research Institute. The patients/participants provided their written informed consent to participate in this study.

## Author contributions

SF: conceptualization, methodology, data analysis, writing – original draft, project administration, and supervision of clinical assessments. SL: conceptualization, methodology, data analysis, writing – review and editing, and project administration. NF: data analysis and writing – review and editing. SS: conceptualization, methodology, writing – review and editing, and project administration. RD: conceptualization, methodology, writing – review and editing, project administration, and supervision of neuropsychological assessment. LK: conceptualization, methodology, supervision, and writing – review and editing. All authors contributed to the article and approved the submitted version.

## Funding

This study was financially supported by Edith Har Esh, Professorship Endowment, New York Medical College, which gave support for assessment materials, subject reimbursement, and article processing.

## Conflict of interest

The authors declare that the research was conducted in the absence of any commercial or financial relationships that could be construed as a potential conflict of interest.

## Publisher’s note

All claims expressed in this article are solely those of the authors and do not necessarily represent those of their affiliated organizations, or those of the publisher, the editors and the reviewers. Any product that may be evaluated in this article, or claim that may be made by its manufacturer, is not guaranteed or endorsed by the publisher.

## References

[1] Lopez-Leon S Wegman-Ostrosky T Perelman C Sepulveda R Rebolledo PA Cuapio A. More than 50 long-term effects of COVID-19: a systematic review and meta-analysis. Sci Rep. (2021) 11:16144. doi: 10.1038/s41598-021-95565-8 34373540 PMC8352980

[2] Premraj L Kannapadi NV Briggs J Seal SM Battaglini D Fanning J. Mid and long-term neurological and neuropsychiatric manifestations of post-COVID-19 syndrome: a meta-analysis. J Neurol Sci. (2022) 434:120162. doi: 10.1016/j.jns.2022.120162 35121209 PMC8798975

[3] Schou TM Joca S Wegener G Bay-Richter C. Psychiatric and neuropsychiatric sequelae of COVID-19 - a systematic review. Brain Behav Immun. (2021) 97:328–48. doi: 10.1016/j.bbi.2021.07.018, PMID: 34339806 PMC8363196

[4] Mazza MG De Lorenzo R Conte C Poletti S Vai B Bollettini I. COVID-19 BioB outpatient clinic study group; Benedetti F. anxiety and depression in COVID-19 survivors: role of inflammatory and clinical predictors. Brain Behav Immun. (2020) 89:594–600. doi: 10.1016/j.bbi.2020.07.037, PMID: 32738287 PMC7390748

[5] Mazza MG Palladini M De Lorenzo R Magnaghi C Poletti S Furlan R. Persistent psychopathology and neurocognitive impairment in COVID-19 survivors: effect of inflammatory biomarkers at three-month follow-up. Brain Behav Immun. (2021) 94:138–47. doi: 10.1016/j.bbi.2021.02.021, PMID: 33639239 PMC7903920

[6] Farooqi M Khan A Jacobs A D’Souza V Consiglio F Karmen CL. Examining the long-term Sequelae of SARS-CoV2 infection in patients seen in an outpatient psychiatric department. Neuropsychiatr Dis Treat. (2022) 18:1259–68. doi: 10.2147/NDT.S357262 35761861 PMC9233564

[7] Kroenke K Spitzer RL Williams JB Monahan PO Löwe B. Anxiety disorders in primary care: prevalence, impairment, comorbidity and detection. Ann Intern Med. (2007) 146:317–25. doi: 10.7326/0003-4819-146-5-200703060-00004 17339617

[8] Blevins CA Weathers FW Davis MT Witte TK Domino JL. The posttraumatic stress disorder checklist for DSM-5 (PCL-5): development and initial psychometric evaluation. J Trauma Stress. (2015) 28:489–98. doi: 10.1002/jts.22059 26606250

[9] PTSD: National Center for PTSD (n.d.). Available at: https://www.ptsd.va.gov/professional/assessment/adult-sr/ptsd-checklist.asp (Accessed May 15, 2023).

[10] Kroenke K Spitzer RL Williams JB. The PHQ-9. J Gen Intern Med. (2001) 16:606–13. doi: 10.1046/j.1525-1497.2001.016009606.x 11556941 PMC1495268

[11] Endicott J Nee J Harrison W Blumenthal R. 1993. Quality of life enjoyment and satisfaction questionnaire: a new measure. Psychopharmacol Bull. (1993) 29:321–6. 8290681

[12] CDC (2022). COVID data tracker Available at: https://covid.cdc.gov/covid-data-tracker/#datatracker-home [Accessed September 26, 2022].

[13] Graf C. The Lawton instrumental activities of daily living scale. Am J Nurs. (2008) 108:52–62. doi: 10.1097/01.NAJ.0000314810.46029.74 18367931

[14] Jackson C. The Chalder fatigue scale (CFQ 11). Occup Med. (2015) 65:86–6. doi: 10.1093/occmed/kqu168 25559796

[15] Randolph C Tierney MC Mohr E Chase TN. The repeatable battery for the assessment of neuropsychological status (RBANS): preliminary clinical validity. J Clin Exp Neuropsychol. (1998) 20:310–9. doi: 10.1076/jcen.20.3.310.823 9845158

[16] Nasreddine ZS Phillips NA BÃ©dirian VÃ© Charbonneau S Whitehead V Collin I. The Montreal cognitive assessment, MoCA: a brief screening tool for mild cognitive impairment. J Am Geriatr Soc. (2005) 53:695–9. doi: 10.1111/j.1532-5415.2005.53221.x 15817019

[17] Chelune GJ Heaton RK Lehman RA. Neuropsychological and personality correlates of patients’ complaints of disability In: . Advances in clinical neuropsychology.Eds. Tarter RE, Goldstein G. Boston, MA: Springer (1996). 95–126.

[18] IBM Corp. IBM SPSS statistics for windows. Armonk, NY: IBM Corp (2020).

[19] Lynch S Ferrando SJ Dornbush R Shahar S Smiley A Klepacz L. Screening for brain fog: is the Montreal cognitive assessment an effective screening tool for neurocognitive complaints post-COVID-19? Gen Hosp Psychiatry. (2022) 78:80–6. doi: 10.1016/j.genhosppsych.2022.07.01, PMID: 35930974 PMC9359801

